# Indirect markers for length adjustment in distal biceps tendon allograft reconstruction

**DOI:** 10.1371/journal.pone.0257057

**Published:** 2021-09-02

**Authors:** Silvan Beeler, Andreas Hecker, Samy Bouaicha, Dominik C. Meyer, Karl Wieser

**Affiliations:** Balgrist University Hospital, Zurich, Switzerland; Boston University School of Medicine, UNITED STATES

## Abstract

Chronic musculotendinous retraction, shortening and fibrosis after distal biceps tendon tears makes a primary reconstruction often difficult or even impossible. Interposition reconstruction with allograft provides a solution, however there is no consensus about appropriate intraoperative graft length adjustment. Therefore, the purpose of this study was to find a practical reference value for distal biceps tendon length adjustment. Three-dimensional surface models of healthy distal biceps tendons were created based on 85 MRI scans. The tendon length was measured from the myotendinous junction to the insertion on the bicipital tuberosity. Inter-epicondylar distance (IED) and radial head diameter (RHD) were measured on antero-posterior radiographs as a surrogate for patient size. Correlations between the tendon length and IED, RHD and patient’s height (PH) were calculated. Mean length of the external part of the distal biceps tendon was 69mm (female 64mm, male 71mm). The tendon length in mm was on average 1.1 times of the IED (mm), 3 times of the RHD (mm) and 0.4 times of PH (cm). Herewith, the tendon length could be predicted within a narrow range of +/-1cm in 84% by using IED, 82% by using RHD and 80% by using PH. Intra- and inter-reader reliabililty of IED and RHD was excellent (R^2^ = 0.938–0.981). The distal biceps tendon length can be best predicted within 1cm with an accuracy of 82–84% using the IED and RHD with an excellent intra- and inter-reader reliability.

## Introduction

Complete ruptures of the distal biceps tendon are relatively frequent and are not always immediately diagnosed [1]. Chronic musculotendinous retraction with early muscle atrophy, shortening and fibrosis may render a primary anatomic repair difficult or even impossible already after few weeks [2]. Untreated, patients may suffer from decreased supination (up to 50%) and flexion (up to 30%) strength of the elbow, fatigue, pain and a “reverse popeye sign” [2–4]. Because non-anatomic repair to the brachial muscle yields inferior results [2, 5], tendon reconstruction to the radial tuberosity is preferred whenever possible.

**Fig 1:**
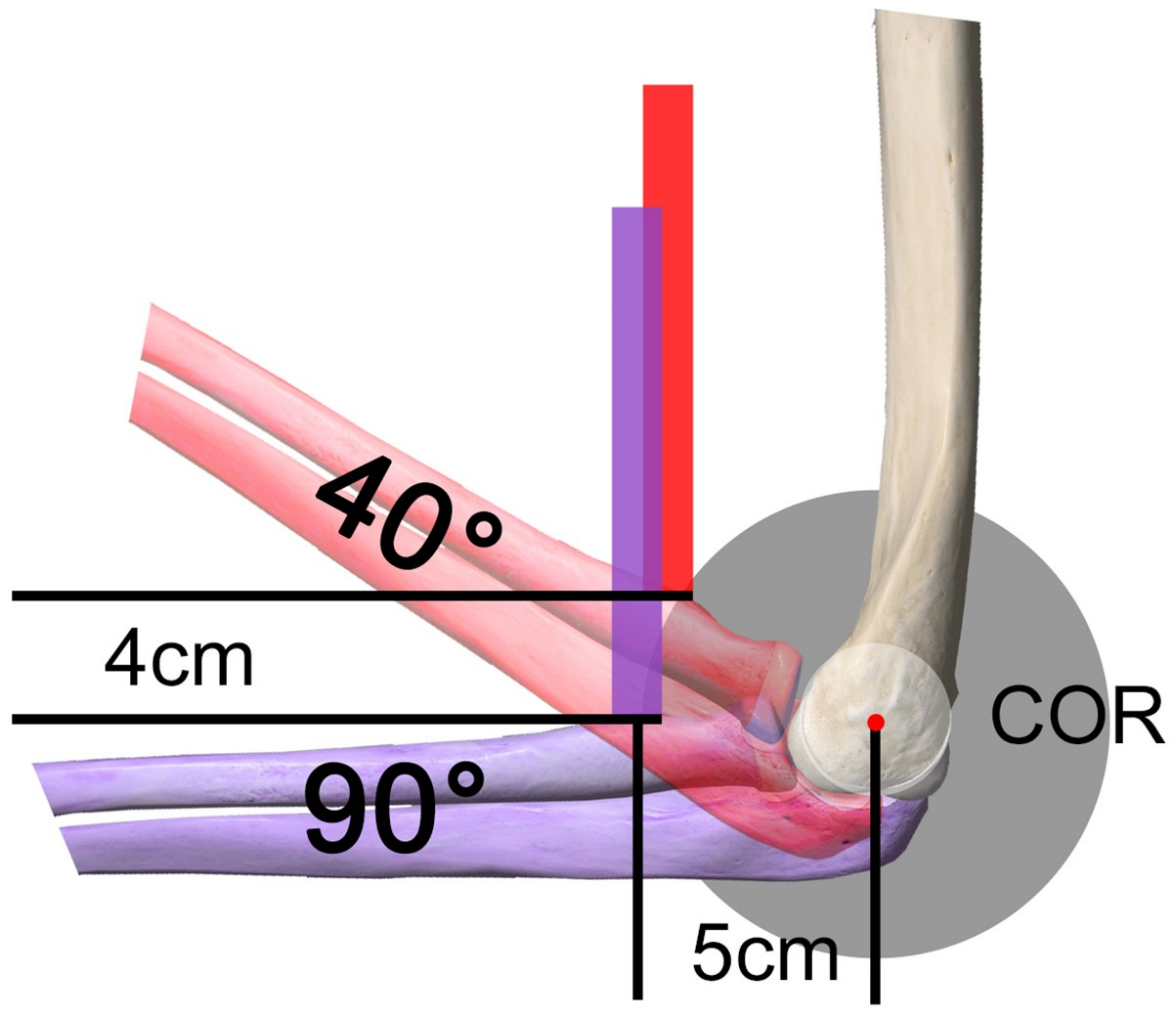
Influence of tendon length by graft fixation in 40° and 90°. Considering that the radial tuberosity moves along a radius of 5cm relative to the center of rotation (COR) of the elbow, an allograft tendon refixation with same traction in 40° (red forearm) compared to 90° (violet forearm) of elbow flexion may lead to a shortening of 4cm, representing almost 60% of the original distal biceps tendon length.

Interposition with an allo- or autograft is advocated if the tendon stump can not be repaired directly to the radial tuberosity with the elbow flexed in 70–90° [6]. The goal is restoration of the original tendon length, providing ideal conditions for good supination and flexion strength, as well as no limitation for elbow extension. So far, there is no consensus in the literature, how the length of a graft reconstruction should be determined intra-operatively or pre-operatively. Recommendations on how to adjust graft tendon length usually rely on the mechanical muscle tension with the elbow flexed in 0° up to 90° [7–10], combined with full supination [8], after removal of the tourniquet [11] or with moderate to maximum [7–11] tendon traction. However, the original anatomical length has not been taken into consideration in this context yet. Considering that the radial tuberosity moves along a radius of 5cm relative to the center of rotation of the elbow, an allograft tendon refixation with same traction in 40° compared to 90° of elbow flexion may lead to a shortening of 4cm, representing almost 60% of the original distal biceps tendon length (Fig 1).

The anatomical structure and tendon architecture of the distal biceps tendon is well known [12–16]. The tendon can be divided into two parts. A visible, external part (external tendon) from the point of muscle fiber termination at the distal myotendinous junction to its insertion on the radial tuberosity and a covered part (internal central tendon) inside the muscle belly. Both, the muscle fiber termination as well as the radial tuberosity can easily be identified intra-operatively and therefore used as landmarks for tendon length measurement.

Therefore, the aim of this study was (1) to develop an applicable 3D MRI measuring tool to measure the length of the (external) distal biceps tendon and (2) to establish an anatomical prediction of the distal biceps tendon length, which might be a helpful tool for intraoperative length adjustment during distal biceps tendon graft reconstruction surgery.

## Materials and methods

### Patients

We retrospectively analyzed all patients without symptoms or known pathology of the distal biceps tendon, which underwent elbow surgery in our hospital between 2010 and 2018. Inclusion criteria was an available MRI scan (1.5 Tesla) with visible distal biceps tendon (from the distal myotendinous junction to radial tuberosity) on axial images, conventional calibrated radiographs (A/P), age of ≥18 years and measured body height. Excluded were all patients with a pathology of the distal biceps tendon (tendinopathy / tear visible on MRI, pain on palpation of distal biceps tendon), surgery of the biceps brachii muscle (proximal or distal), extension/flexion contractions of the elbow and malunion/osteoarthritic changes of the radial head and epicondyles. All MRI scans were performed in superman position (prone position with the arm extended over the head). Ethical approval was granted by Kantonale Ethikkommission of Zurich, Switzerland (BASEC-Nr. 2019–00286), and informed written consent was obtained from all individual participants included in the study.

Of all 507 operated patients, 85 elbows of 79 patients were included. Reasons for surgery were epicondylopathy (63), instability (8), tumor (3) and others (11). Demographics of all patients are listed in Table 1.

**Table 1:** Demographics.

Number of patients / elbows	79 / 85 (6 bilateral)
Age	mean 45.5 years, range 18–81, SD 12.4
Gender	31 women, 54 men
Side	50 right, 35 left
Height	mean 173cm, range 149-193cm, SD 9.09

### Measurements

#### Distal biceps tendon length measurement

**Fig 2:**
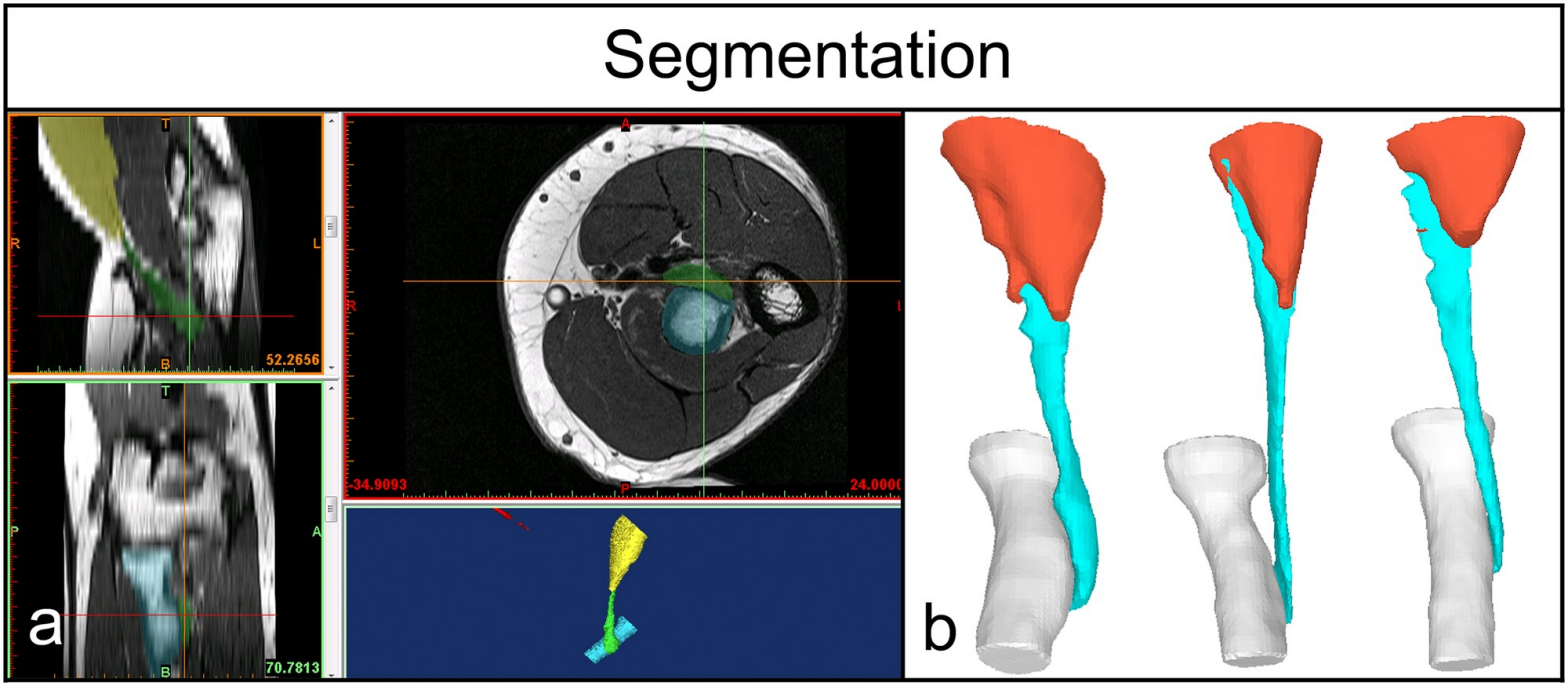
Segmentation. a) Manual segmentation of the distal biceps tendon, proximal radial bone and distal muscle belly of the brachial biceps. b) Generation of 3D surface models: Three examples.

**Fig 3:**
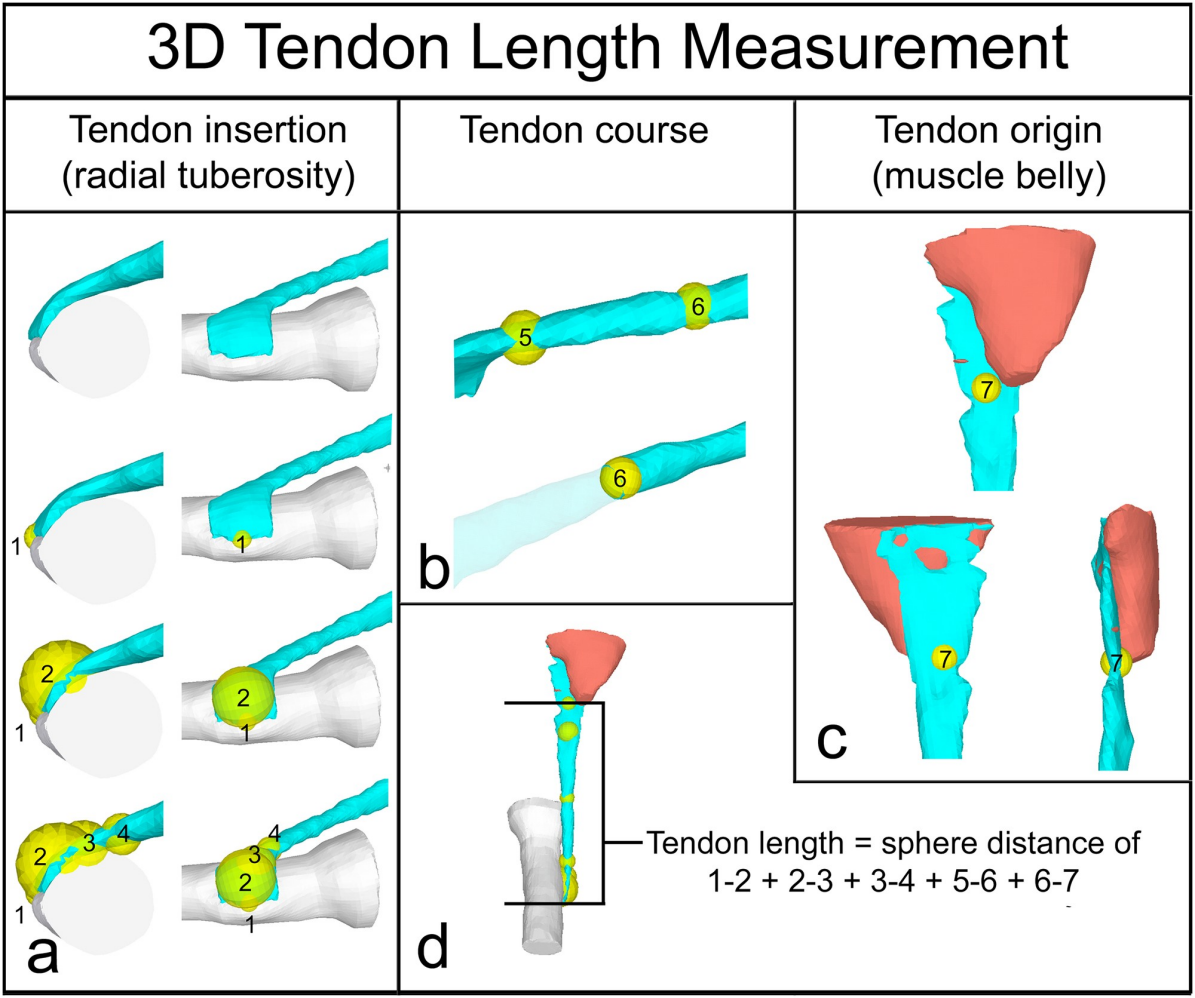
3D tendon length measurement. 3D tendon length measuring from the distal myotendinous junction to the insertion on the bicipital tuberosity: a) Tendon insertion (radial tuberosity): The first sphere was placed on the tendon insertion, centered on the radial tuberosity. b) Tendon course: 4–8 further spheres were placed along the entire tendon, wherever the tendon changed the direction. c) Tendon origin (muscle belly): The last sphere was placed at the distal myotendinous junction. d) Tendon length measuring: The tendon length was measured as the distance of the center of the sphere 1–2 + 2–3 + 3–4 + 5–6 + 6–7.

- 3D surface models: Segmentation of the distal biceps tendon, distal biceps muscle belly and proximal radial bone were performed manually by two trained orthopaedic surgeons on axial MRI images (T1) with the Materialise Interactive Medical Control System (MIMICS) 3D reconstruction software program 18.0 (Materialise, Leuven, Belgium). Slice thickness was on average 3.2mm (2.5–4.0mm). The 3D surface models were smoothed (gap closing distance 0.0mm, smallest detail 1.0mm) using the wrapping functionality of the software and exported as a Staroffice Template Drawing (.std) file. (Fig 2)
- 3D tendon length measurement: The length of the distal biceps tendon was defined as the external tendon part from the point of muscle fiber termination (distal myotendinous junction) to the insertion on the bicipital tuberosity. The 3D surface models were imported in the in-house developed software program CASPA (Computer Assisted Surgery Planning Application, Balgrist CARD AG). 3D spheres were placed along the entire tendon. The first sphere was placed on the tendon insertion, centered on the radial tuberosity. Further spheres where placed throughout the whole course of the tendon, each centered on the tendon. The more curved the measured tendon section was–due to the pronated radial tuberosity and displacement by the brachial muscle–the more spheres where applied to reproduce the original tendon length as anatomically as possible. The last sphere was placed at the distal myotendinous junction. On average, there were 7–8 spheres used (min 6, max 10). (Fig 3)

#### Individual parameters

**Fig 4:**
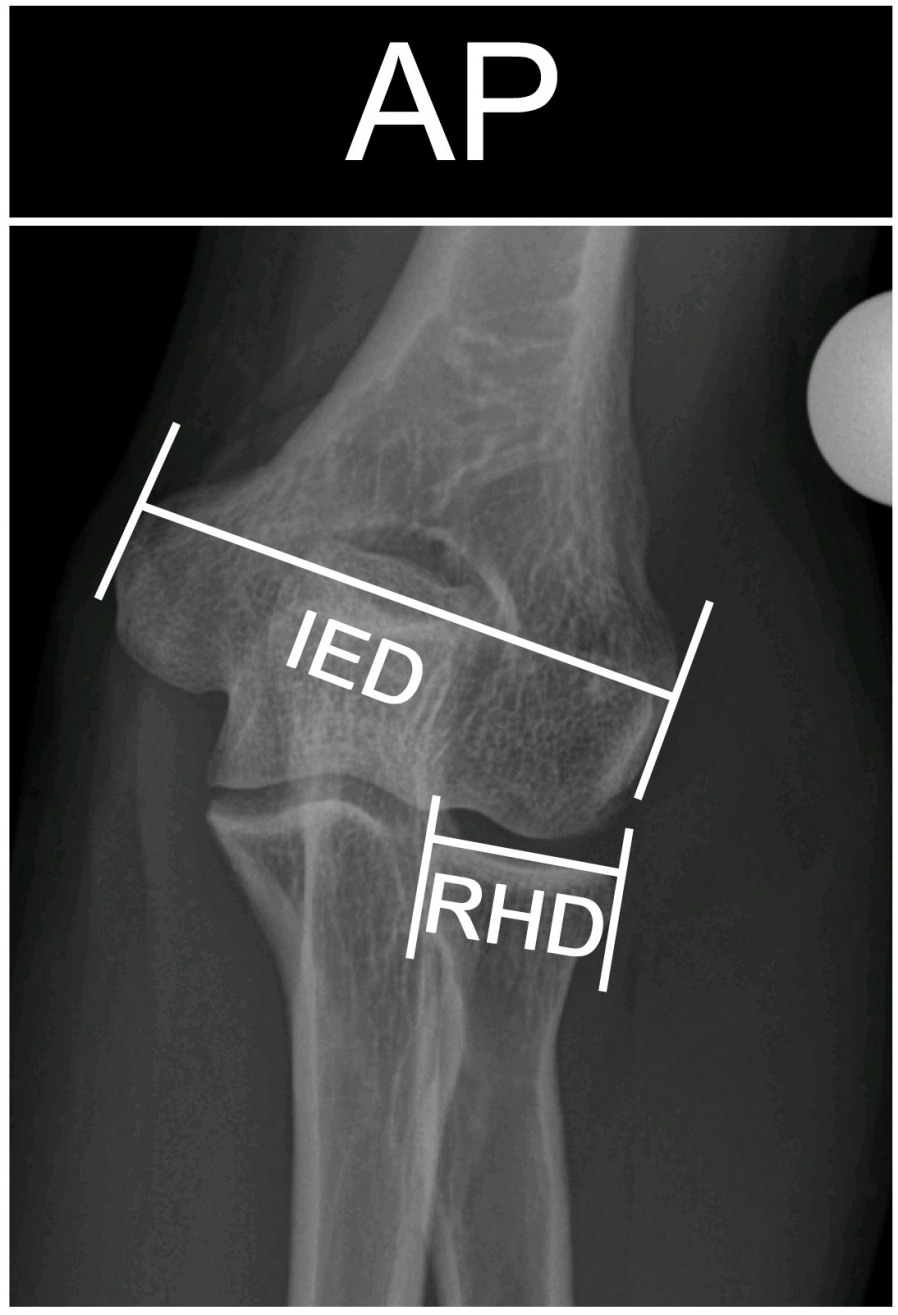
Inter-epicondylar distance (IED) and radial head diameter (RHD). IED = inter-epicondylar distance: Measured from the medial to the lateral epicondyle. RHD = radial head diameter: Measured as the diameter of the radial head, perpendicular to the radial head surface.

- Patient’s height (PH): was measured in cm in standing position (without shoes) and is routinely done in our pre-operative patient assessment in a standardized manner.
- Inter-epicondylar distance (IED): was measured in A/P radiographs, from the medial to the lateral epicondyle. All radiographs were first calibrated based on a 25mm calibration ball with the mediCAD^®^ 5.0 planning software (mediCAD Hectec GmbH). (Fig 4)
  The assessment of reproducibility of the IED was calculated for the same reader (intra-reader reliability of the same radiograph; repeated measurement after 1 month) and for two different readers (inter-reader reliability of the same radiograph) on all 85 elbows.
- Radial head diameter (RHD): was measured in calibrated A/P radiographs, as the largest radial head diameter perpendicular and about 3mm distal to the radial joint surface. (Fig 4). The assessment of reproducibility was performed in the same way as above.

### Statistics

Statistical analysis was performed with SPSS (IBM Corp. IBM SPSS Statistics for Windows, Version 24.0. Armonk, NY: IBM Corp.). Descriptive analysis and independent sample test was performed to investigate patient’s characteristics and differences between gender and side. A p-value of <0.05 was considered to be statistically significantly different. Histograms were generated for better visualization of our data. The reproducibility of IED and RHD measurement was assessed with the inter-class correlation coefficient (ICC). Pearson correlation coefficient was used to calculate the relationship of patient specific size parameters to the tendon length.

## Results

**Fig 5:**
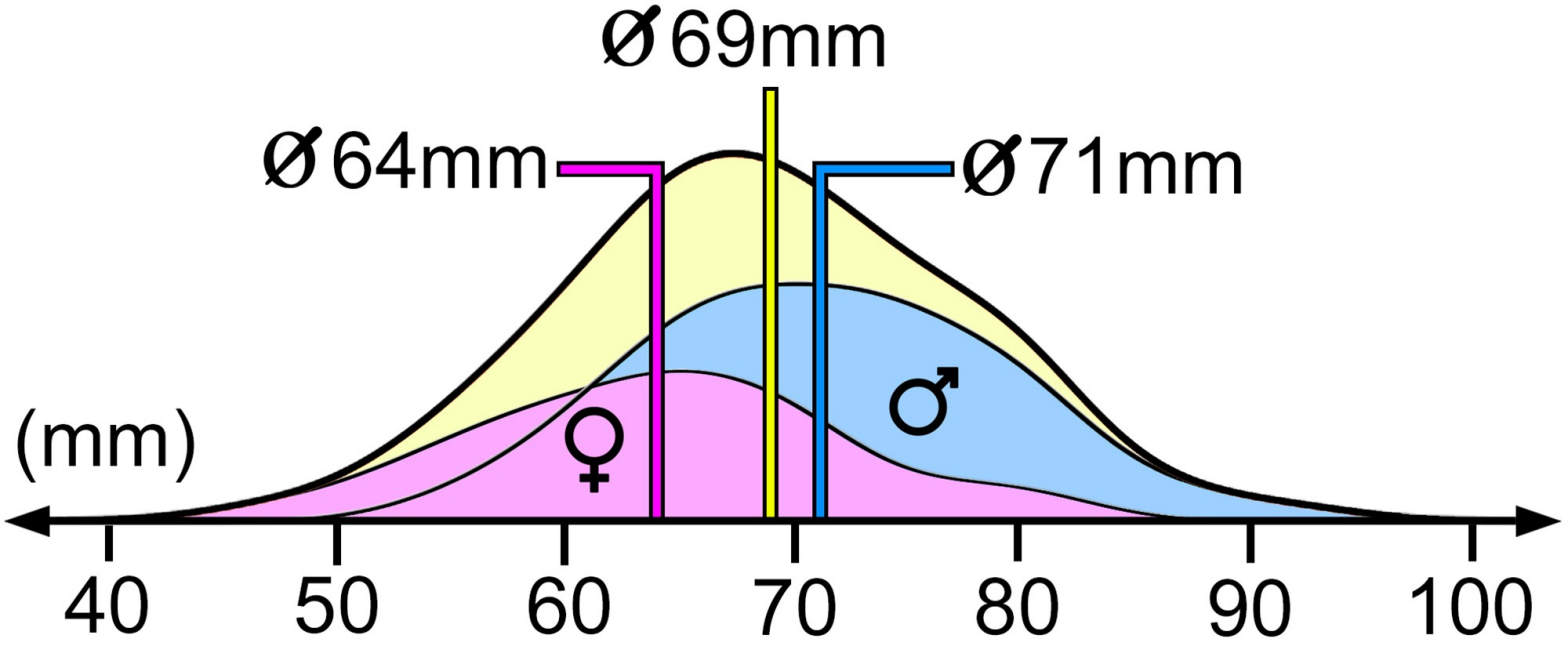
Tendon length and gender. This histogramm shows the distribution of the tendon length in general (yellow curve; mean value of 69mm), in women (violett; mean value of 64mm) and in men (blue; mean value of 71mm).

Mean distal biceps tendon length (TL) was 69mm (SD+/-6.7mm) and on average 7mm shorter in women (p<0.001) (Fig 5). Furthermore, women were on average 12cm smaller and had a 9mm shorter IED, respectively 4mm shorter RHD (all p<0.001). Interestingly, the size corrected TL (TL divided by IED, RHD or PH) was no longer different between women and men. In addition, there was no difference between right and left side (p = 0.264). All values of TL, IED and RHD can be found on Table 2 and the influence of gender and side is shown in Table 3.

**Table 2:** Basic dimensions.

**TL**	Mean 69mm, SD 8.67, Range 45mm (min 47mm, max 92mm)
**IED**	Mean 63mm, SD 6.04, Range 24mm (min 50mm, max 74mm)
**RHD**	Mean 23mm, SD 2.15, Range 9mm (min 19mm, max 28mm)

TL = Tendon Length of the distal biceps, IED = inter-epicondylar distance, RHD = radial head diameter

**Table 3:** Measurments and correlations in dependence of gender and side.

**Gender**			
	**Women**	**Men**	**p-value**
**TL**	64mm, (SD 8.1, min 47mm, max 81mm)	71mm (SD 7.9, min 55mm, max 92mm)	<0.001
**IED**	57mm (SD 4.6, min 50mm, max 67mm)	66mm (SD 4.0, min 57mm, max 74mm)	<0.001
**TL/IED**	1.13 (SD 0.167, min 0.747, max 1.51)	1.09 (SD 0.107, min 0.838, max 1.35)	0.106
**RHD**	21mm (SD 1.6, min 19, max 25)	25mm (SD 1.4, min 22, max 28)	<0.001
**TL/RHD**	3.02 (SD 0.388, min 2.04, max 3.88)	2.92 (SD 0.308, min 2.10, max 3.83)	0.162
**PH**	165cm (SD 7.3, min 149cm, max 177cm)	177cm (SD 7.1, min 164cm, max 193cm)	<0.001
**TL/PH**	0.389 (SD 0.048, min 0.272, max 0.491)	0.404 (SD 0.045, min 0.319, max 0.547)	0.147
**Age**	44.3y (SD 11.8, min 19y, max 70y)	46.2y (SD 12.8, min 18y, max 81y)	0.498
**Side**			
	**Right**	**Left**	**p-value**
**TL**	70mm (SD 8.6, min 54mm, max 92mm)	68mm (SD 8.7, min 47mm, max 83mm)	0.264
**IED**	63mm (SD 5.9, min 50mm, max 74mm)	62mm (SD 6.2, min 51mm, max 74mm)	0.544
**RHD**	23mm (SD 2.2, min 19mm, max 28mm)	23mm (SD 2.2, min 19mm, max 27mm)	0.765
**PH**	173cm (SD 8.7, min 155cm, max 193cm)	172cm (SD 9.8, min 149cm, max 193cm)	0.523
**Age**	45.9y (SD 11.5, min 18y, max 70y)	44.8y (SD 13.7, min 19y, max 81y)	0.692

TL = Tendon Length of the distal biceps, IED = inter-epicondylar distance, RHD = radial head diameter, PH = patient’s height

Overall, the radiographic parameters (RHD; R = 0.483, IED; R = 0.470) showed a better correlation to the individual distal biceps tendon length than PH (R = 0.393). The tendon length was on average 1.1 times the size of the IED in mm (= 69mm/63mm), 3 times of the RHD in mm (= 69mm/23mm) and 0.4 times of PH in cm (= 69mm/173cm).

**Fig 6:**
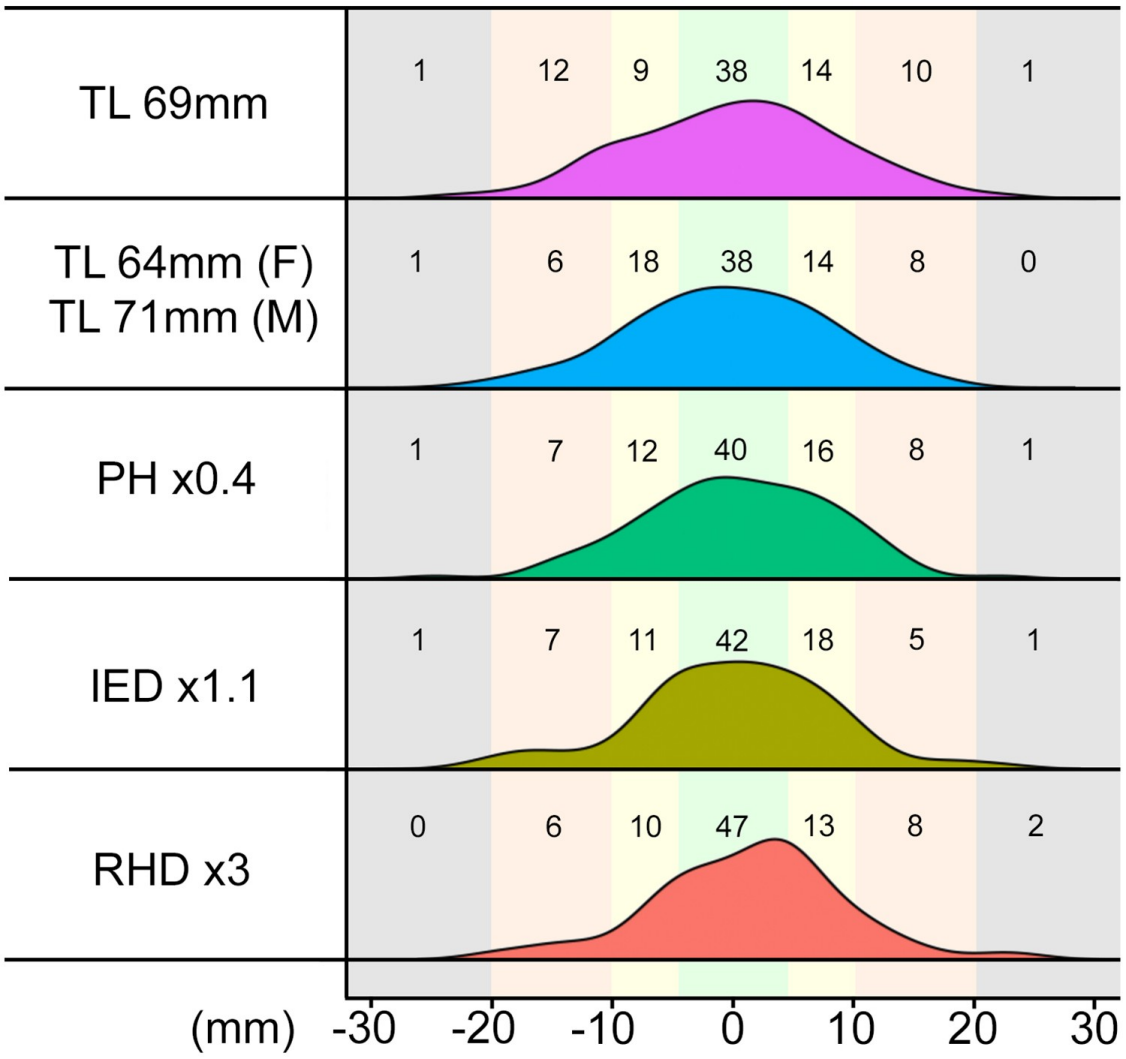
Distal biceps tendon length prediction by. This histogram shows the tendon length prediction by mean tendon length (TL; mean value 69mm), mean tendon length with gender distinction (TL; F Female mean value 64mm, M Male mean value 71mm), patient’s heigth (PH in cm x0.4), inter-epicondylar distance (IED in mm x1.1) and radial head diameter (RHD in mm x3). The scale reaches from 0 to -30mm (left) and +30mm (right). Perfect match would be a value of 0. And positive values imply that the calculated tendon assumes a too long, negative values too short. For example, the calculated tendon by RHD tends to be rather longer (23 elbows >+0.5cm) than smaller (16 elbows <-0.5cm).

The distal biceps tendon length could be predicted within +/-1cm with an accuracy of 72% by mean tendon length (TL), 82% with gender correction, 80% by PH, 84% by IED and 82% by RHD. All values can be found on Table 4 and Fig 6.

**Table 4:** Distal biceps tendon length prediction by.

	% ≤+/-0.5cm	% ≤+/-1.0cm	% ≤+/-2.0cm
Mean tendon length (TL) 69mm	38 (= 45%)	61 (= 72%)	83 (= 98%)
Mean tendon length (TL) 64mm (F) / 71mm (M)	38 (= 45%)	70 (= 82%)	84 (= 99%)
Patient’s height (PH) (cm) x0.4	40 (= 47%)	68 (= 80%)	83 (= 98%)
Inter-epicondylar distance (IED) x1.1	42 (= 49%)	71 (= 84%)	83 (= 98%)
Radial head diameter (RHD) x3.0	47 (= 55%)	70 (= 82%)	82 (= 96%)

The anatomical length of the distal biceps tendon was predicted by mean tendon length (TL), TL with gender distinction, patient’s heigth (PH), inter-epicondylar distance (IED) and radial head diameter (RHD). The first row shows the number and percentage of tendons which could be predicted within -0.5mm and +0.5mm of the original length. The second row within -1.0cm and + 1.0cm and the third row within -2.0cm and +2.0cm.

Intra- and inter-reader reliability of IED and RHD on A/P radiographs was excellent (Intra-reader reliability IED 0.996, RHD 0.992, inter-reader reliability IED 0.988, RHD 0.981).

## Discussion

To our knowledge, this is the first study using anatomical bony markers to predict the original distal biceps tendon length. Of all five tested methods, IED and RHD were most reproducible and were able to reach an accuracy of +/-1cm in 85%, respectively 82% of the elbows. In addition, the radiological intra- and inter-observer correlations were extraordinarily high with 0.99 each.

Overall, our anatomical findings in MRI are comparable to the existing reports [12–16]. The external tendon part was 6.9cm, compared to 5.7–9.2cm in the literature. However, not only previous reports, but especially our investigation revealed a high variation of the tendon length of up to 45 mm between individuals. So far, no anatomical or radiological markers to predict the distal biceps tendon length have been evaluated yet. Overall, we found only five anatomical studies with more than 10 cadavers. In the largest series with 50 cadavers, Joshi found an average tendon length of 7.6cm and no side difference [14]. But he did not distinguish between sex and body size, as almost all other anatomical studies [12, 13, 15]. Only Shastry et al. differentiated between 10 female and 14 male cadavers [16]. They found no side-to-side difference and females had a 7mm longer tendon on average, but not statistically significantly. In our analysis, gender and body size were correlated to the tendon length and compared between each other. Females had a 7mm shorter tendon and where 12cm shorter in body height. However, after size adjusted tendon length, there was no significant difference between sexes, which corresponds to the study of Shastry [16].

The main goal of this investigation was to provide a useful tool for length adjustment of the biceps tendon during tendon interposition reconstructions in case of chronic irreparable biceps tendon tears. However, the clinical significance of a lengthened or shortened distal biceps tendon is unclear and some reports highlight its potential influence on patients elbow strength. Nielsen et al. published a case report in 1987 about a patient with a traumatic rupture of the lacertus fibrosus and resultant distal biceps tendon elongation with resulting weakness of elbow flexion and supination [17]. After reconstruction, the weakness could be restored. Marshall et al. marked intraoperatively 11 repaired distal biceps tendons with tantalum beads and could show a mean tendon lengthening after primary tendon reconstruction of about 23mm (11-31mm) after 16 weeks [18]. Tendon lengthening was associated with a greater loss of supination strength.

We identified several retrospective outcome studies with low numbers of patients and a great variety of surgical techniques and graft choices. Some authors are afraid of over-tensioning [7, 19] with resultant extension deficit and others want to avoid under-tensioning [20]. While primary distal biceps tendon repairs have good clinical results [21], chronic retraction with the need of allograft interposition leads more frequently to postoperative weakness, muscle belly cramping and flection contractions [10, 22]. Although the clinical evidence of inadequate tendon length adjustment is not proven to be the cause of inferior results, one can assume that optimal preload with anatomically restored myotendinous length (i.e. isometry) is an elementary factor for satisfying force development. In order not to be solely dependent on the surgeons experience for the intra-operative tendon length adjustment, quantitative a reference values are mandatory.

A further theoretical concern of graft length adjustment is, that even if the original tendon length could be perfectly restored, a postoperative allo- or autograft tendon lengthening might occur. In a biomechanical study for anterior cruciate ligament reconstruction, achilles tendon allograft elongation averaged by 1.4% +/-1.6% after cyclic testing with 1000 submaximal cycles in the physiological loading between 50 N and 250 N was shown. Based on an average tendon length of 7cm, there would be only an elongation of 0.98mm and may be even smaller in elbow due to the lower cycling loadings [23].

Nevertheless, intraoperative graft length when performing a distal biceps reconstruction always depends on the clinical situation encountered, not necessarily on the anatomic norms. Therefore, the calculated tendon length is merely a guide and must be adjusted to suit the individual intraoperative situation.

**Fig 7:**
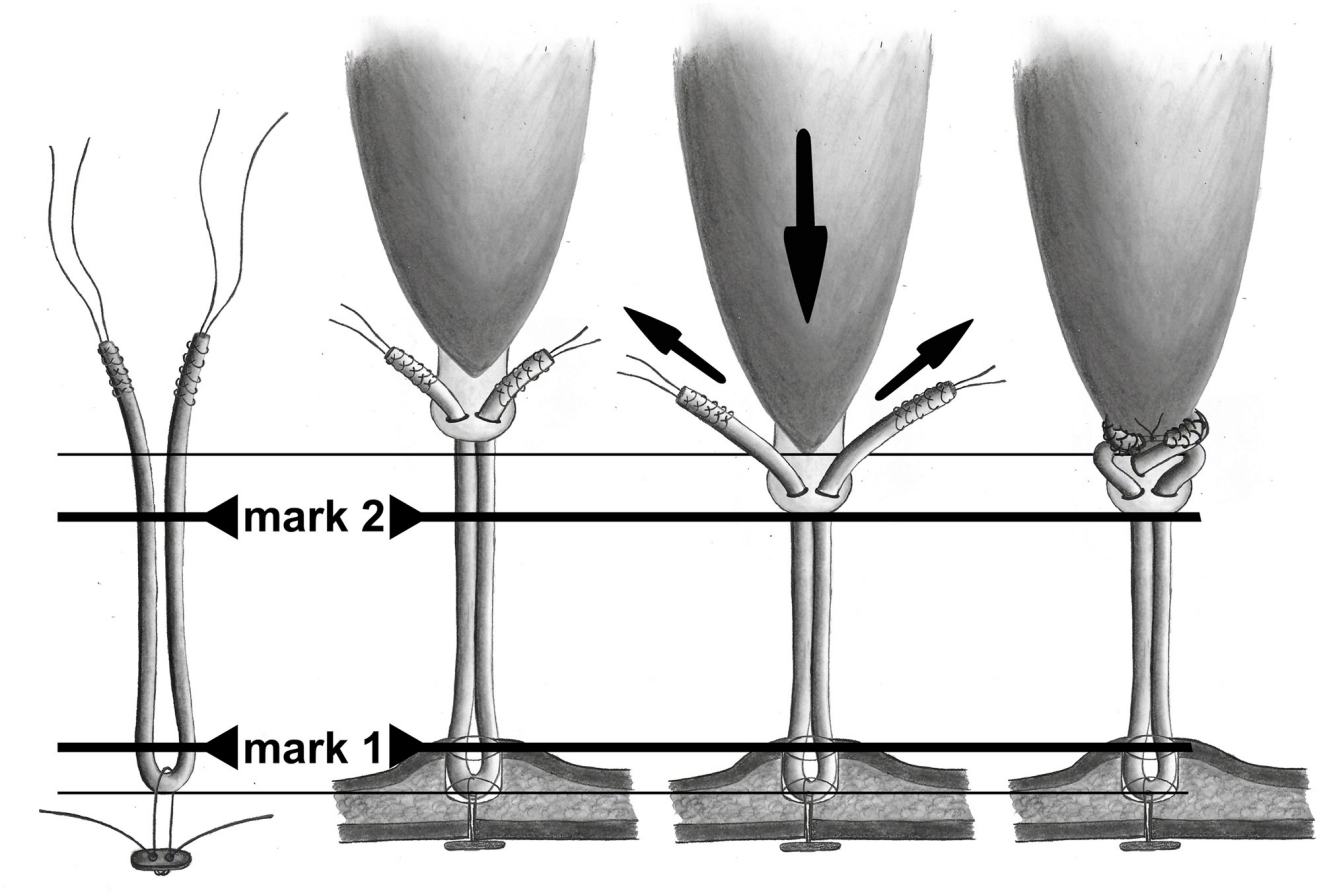
Surgical technique for distal biceps tendon auto-/allograft length adjustment. a) Tendon graft: Both graft ends are reinfored by #2 FiberWire. The bone tunnel length (mark 1) and calculated tendon length without the remaining tendon stump (mark 2) are marked on the graft. b) Tendon graft fixation distally: The tendon graft is first fixed distally and shuttled through the biceps tunnel. After that, the sutures are penetrated throught the remaining tendon with a sharp suture grasper clamp. c) Tendon length adjustment: The elbow is flexed as far as needed and the graft tensionned, until the marker is flash with the remaining tendon end. A slide back of the tendon is preventionned by some sutures (#2 Vicryl). d) Tendon graft fixation proximally: The graft is interwaved through the remaining tendon–two to three times, depending on graft length–and finally knotted together. Further sutures (#2 Vicryl) are performed.

**Fig 8:**
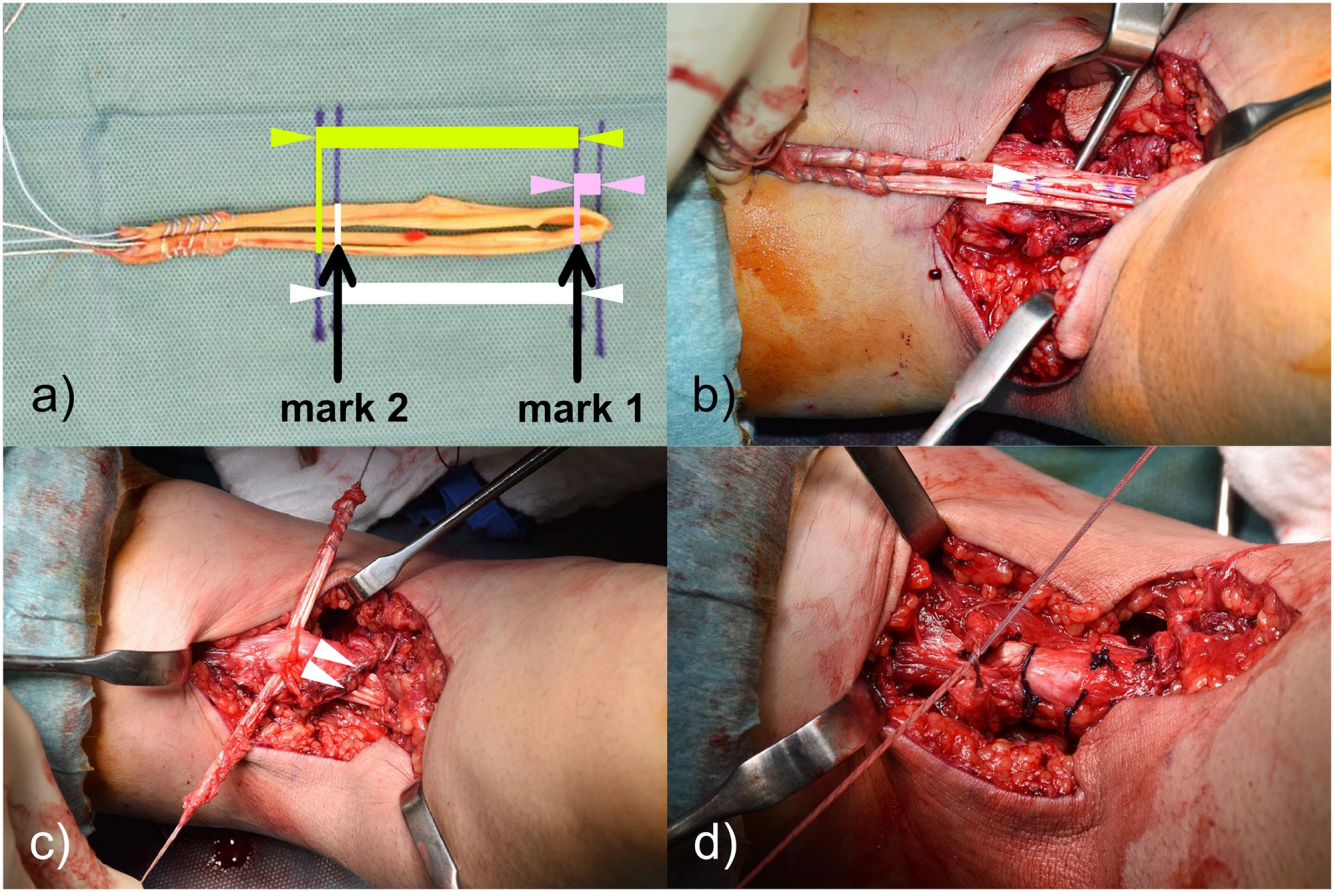
Intraoperative pictures of graft interposition with a hamstring autograft. a) Green = calculated tendon length, White = calculated tendon length without remaining tendon stump, Pink = intraosseous distance of 0.5cm. Mark 1 = corresponding to intraosseous distance, Mark 2 = corresponding to calculated tendon length without remaining tendon stump. b) The arm is flexed, mark 2 visible. c) Tendon length adjustment. d) Interwave of the autograft.

In our clinic, all patients with symptomatic distal biceps tendon tears receive standardized radiographs in two planes (A/P and lateral) and an MRI scan in superman position. The required tendon length can be determined preoperatively by IED and/or RHD. If primary reconstruction is not possible even with a 90° flexed elbow, we use an ipsilateral semitendinosus (+/- gracilis) tendon as an interposal autograft. The graft is used doubled and distally fixed by a cortical button technique. We add two marks on the graft with a sterile pencil. The first mark corresponds to the intraosseous distance. And the second mark to the calculated tendon length without remaining tendon stump length (Figs 7a and 8a). The cortical button can now be inserted bicortical, flipped on the opposite cortex, the graft retracted and fixed in the bone tunnel. The first mark should now be flush with the cortex and the graft can be pulled through the soft tissue tunnel (Fig 8b). In a next step, the two graft ends are penetrated through the remaining tendon stump with a thin, pointed suture grasper clamp (Figs 7b and 8c). The elbow can now be flexed as long as the graft can be retracted to the second mark (calculated tendon length without remaining tendon) (Figs 7c and 8c). Some stitches (#2 Vicryl Ethicon) prevent a slide back of the graft. This procedure has to be repeated two to three times and the tendon ends finally knotted together (Figs 7d and 8d).

In summary, to our best knowledge, we describe the most comprehensive series regarding the distal biceps tendon length and possible predictive factors for it, however, we are aware of some limitations of this investigation. First, there is no clinical proved correlation, that 10mm is an appropriate threshold for graft length estimation. And with a threshold of 5mm, the tendon length could be estimated only in about half of the cases. In the end, this limitation can only be answered by the clinical application in further studies. Second, our results are based on 85 elbows of 79 patients. Although, this is the largest series to date, there could be differences between race and our results may only be valid for people of European descent. Third, there are some disadvantages of 3D tendon measurement on MRI scans compared to direct measurement on cadavers. Inaccuracy could appear due to thick MRI slides or curved tendon courses. Our MRI slide thickness was however relatively thin with on average 3.2mm and some inaccuracy may only be relevant for determination of the muscle belly. The radial tuberosity lies parallel to the axial slides and therefore easily visible even with thicker slides. However, these limitations are compensated by the advantages of 3D MRI tendon measurement. A much larger number can be included as well as important demographic data, such as age, body height and health status.

Finally, our measurement method is not validated yet. However, intra-reader and inter-reader reliability was very high, suggesting the presented method to be very reproducible.

## Conclusion

The average tendon length of the distal brachial biceps is 69mm with no side-to-side differences. Body size influences the tendon length more than sex. The distal biceps tendon length can be predicted within 1cm with an accuracy of 82–84%, by calculating 1.1x of the IED or 3.0x of the RHD with an excellent intra- and inter-reader reliability. IED and RHD could therefore be a helpful reference value for intraoperative tendon length adjustment in distal biceps reconstruction using auto- or allograft tendons.

## Supporting information

S1 Dataset (XLSX)

## Abbreviations

3D: three-dimensional
A/P: antero-posterior
ICC: Interclass correlation coefficient
IED: Inter-epicondylar distance
MIMICS: Materialise Interactive Medical Control System
MRI: magnetic resonance imaging
PH: Patient’s heigth
RHD: Radial head diameter
SD: Standard deviation
TL: Tendon length
